# Effect of Extrusion Process on Microstructure, Corrosion Properties, and Mechanical Properties of Micro-Alloyed Mg–Zn–Ca–Zr Alloy

**DOI:** 10.3390/ma17174263

**Published:** 2024-08-28

**Authors:** Zemin Yu, Wenxin Hu, Zhiqiang Chen, Lei Shi, Lei Yang, Jianfeng Jin, Erlin Zhang

**Affiliations:** 1 State Key Laboratory of Baiyunobo Rare Earth Resource Researches and Comprehensive Utilization, Baotou Research Institute of Rare Earths, Baotou 014030, China; zeminyu_1700@brire.com (Z.Y.); chenzhiqiang_brire@163.com (Z.C.); stonesir@shu.edu.cn (L.S.); 2 Key Laboratory for Anisotropy and Texture of Materials, Education Ministry of China, School of Materials Science and Engineering, Northeastern University, Shenyang 110819, China; jinjf@atm.neu.edu.cn (J.J.); zhangel@atm.neu.edu.cn (E.Z.); 3 Research Center for Metallic Wires, Northeastern University, Shenyang 110819, China

**Keywords:** Mg–Zn–Ca–Zr alloy, extrusion, Zr-rich phase, corrosion properties, mechanical properties

## Abstract

The effect of the extrusion process on the microstructure, corrosion, and mechanical properties of Mg–Zn–Ca–Zr alloy has been investigated. Zn and Ca were both in a solid solution and only the Zr-rich phase was observed in the homogenized and extruded alloys. The Zr-rich phase was obviously refined after extrusion. The corrosion rate of the homogenized alloy decreased by about 25% after extrusion. This is because the refined Zr-rich phase was easier to cover with the deposited corrosion products, which reduced the cathodic reaction activity of the Zr-rich phase. The corrosion rate is similar for the alloys extruded at 320 °C and 350 °C since the size and distribution of the Zr-rich phase were not different in the two conditions. The alloy extruded at 320 °C has a smaller grain size and better comprehensive mechanical properties.

## 1. Introduction

The excellent biomechanical properties, degradability, and good biocompatibility of magnesium alloy have made it a widely studied material with promising potential for medical applications [1,2,3,4,5]. However, its application is limited due to low mechanical properties and poor corrosion resistance in liquid environments [6,7,8,9].

Alloying is an effective way for enhancing the corrosion resistance and mechanical properties of magnesium alloys [10]. Among various alloy systems, Mg–Zn–Ca alloy shows great potential as an implantable medical material [11,12]. Bakhsheshi Rad et al. [13,14] proposed that in a Mg–xCa alloy system, when the Ca content exceeds 0.5 wt.%, an increase in Ca content leads to a higher content of Mg_2_Ca phase, which acts as an anode phase and increases the corrosion rate of magnesium alloy. In Mg–xZn and Mg–0.8Ca–xZn alloy systems, increasing Zn content also results in a higher corrosion rate due to its influence on the type and distribution of second phases within the alloy caused by the Zn/Ca ratio. Zheng et al. [15] proposed that the content of Ca should be controlled in the range of 0.6~1.0 wt.%, otherwise a ternary Ca_2_Mg_6_Zn_3_ phase and binary Mg_2_Ca phase may be formed in the Mg–Zn–Ca alloy, which will form micro-galvanic couples with the magnesium matrix, resulting in an increased corrosion rate in the alloy. In extruded Mg–xCa alloys, yield strength increases with increasing Ca content while elongation gradually decreases [16]. In extrusion and rolling-processed Mg–xZn–Ca alloys, both yield strength and elongation increase with rising Zn content [11,17]. In addition, the addition of zirconium element not only can refine the grain size of magnesium alloy [18,19] but also can remove iron impurities in magnesium alloy to improve corrosion resistance [20] since iron impurities can significantly reduce the corrosion resistance of magnesium alloy [21,22,23,24]. However, a higher Zr content may lead to the formation of Zr particles in the magnesium alloy, which is unfavorable for the corrosion resistance of the magnesium alloy [18,25,26]; it is necessary to control Zr content reasonably. Thus, we have fabricated a micro-alloyed Mg–Zn–Ca–Zr alloy for medical applications.

Extrusion has always been considered an effective method for preparing high-strength magnesium alloys [27,28]. Additionally, extrusion also influences the corrosion resistance of magnesium alloys [29]. Some literature reported that extrusion could improve corrosion resistance [30,31,32,33,34,35,36,37], while others reported the opposite [38,39]. For example, Marodkar et al. [36] reported that due to the absence of porosity, refined microstructure, and some amount of dissolved β-Mg_17_Al_12_ in the matrix region, an extruded AZ91 alloy exhibited superior corrosion resistance compared to gravity-die-casting and squeeze-casting AZ91 alloy. Song et al. [38] investigated the effect of microstructure changes on the corrosion behavior of equal-channel-angular-pressed (ECAPed) pure magnesium. It is found that ECAPed pure magnesium is more corrosive than cast pure magnesium, which is due to more rapid passive films that present no improved protection and the mass of energetic crystalline defects that drastically activate the corrosion reaction. In the extrusion process, the extrusion temperature generally plays an important role in affecting both corrosion and mechanical properties. Li et al. [40] reported that both the mechanical properties and the corrosion properties of Mg–2Nd–0.2Zn alloy decreased with increasing extrusion temperature. Zhang et al. [41] obtained similar results when investigating the impact of extrusion temperature on the mechanical properties and corrosion rate of Mg–3Nd–0.2Zn–0.4Zr alloy. Many scholars believe that higher extrusion temperatures result in a decrease in corrosion rate [42,43,44]. However, Mohammadi et al. [45] discovered that in Mg–5Zn–1Y–xCa alloys, the correlation between extrusion temperature and corrosion rate is not always consistent across different calcium content levels. Therefore, it is crucial to study the influence of the extrusion process on the corrosion and mechanical properties of Mg alloys.

This work investigates the impact of the extrusion process on the microstructure, corrosion behavior, and mechanical properties of Mg–0.5Zn–0.3Ca–0.03Zr alloy, with the aim of developing a Mg–Zn–Ca–Zr alloy with superior comprehensive mechanical and corrosion properties for medical applications.

## 2. Experimental Procedures

### 2.1. Preparation and Characterization of Materials

The raw materials for preparing the alloy were commercial pure magnesium (99.95 wt.%), high-purity zinc (99.99 wt.%), Mg-30 wt.% Ca master alloy, and Mg-30 wt.% Zr master alloy. The alloy was melted in a low-carbon steel crucible at 700 °C in an electric resistance furnace. After melting, the melt was stirred for 2 min and then held for 10 min. Finally, the melt was poured into a steel mold coated with boron nitride and preheated to 200 °C. The composition was determined by optical emission spectroscopy (Spectroscopy Laboratory M9, SPECTRO, Kleve, Germany) and inductively coupled plasma atomic emission spectrometry (Perkin-Elmer Optima 8300, Waltham, MA, USA). The results are shown in Table 1.

The as-cast alloy was heat-treated at 350 °C for 12 h plus at 450 °C for 10 h and then quenched in cold water. The alloy subjected to homogenization heat treatment was named HT-ZXK. The alloy obtained by homogenization heat treatment was extruded at 320 °C and 350 °C to obtain extruded rods with a diameter of φ15mm, which were named 320-E and 350-E, respectively. The extrusion ratio was 30 and the extrusion rate was 0.5 mm/s.

The optical microstructure was observed using a metallographic microscope (OLYMPUS GX71, Shinjuku, Japan), and the average grain size of the sample was statistically analyzed using the intercept method in Image-pro Plus (6.0) software. The metallographic samples were ground to 2000 mesh and polished using 0.5 µm diamond polishing paste. The microstructure of the alloy was further characterized using a Zeiss Ultra 55 (Oberkochen, Germany) scanning electron microscope (SEM) equipped with energy-dispersive X-ray spectroscopy (EDS).

### 2.2. Phase Diagram Calculation

The relevant phase diagrams of the alloying system were calculated using the thermodynamic software Pandat (2021) and the PanMg2021 database.

### 2.3. Corrosion Tests

Corrosion tests were conducted in a thermostatic water bath (37 ± 0.5 °C) using SBF solution [2]. The sample size for ZXK alloy was always 10 mm × 10 mm × 10 mm, and for extruded alloy, it was φ15 × 4 mm. Each side of the sample was mechanically polished with 2000 grit emery paper, and the initial mass was measured. After 21 days of immersion, corrosion products were removed by immersing specimens in 180 g/L chromic acid for 20 min at room temperature. The weight of the specimens was measured, and the macro corrosion morphology was recorded. At least three specimens were measured for each type of the studied alloys. The corrosion rate (mm/year) was calculated using the following equation:

(1) $${\mathrm{CR}}_{\mathrm{wl}}=\frac{8{.76\;\times \;10}^{4}\;\times \;\Delta g}{A\;\times \;t\;\times \;\rho }\;$$

In Equation (1), Δg is loss of weight (g); A is area of the surface (cm^2^); t is time of immersion (h); and ρ is material density (g·cm^–3^). Three specimens for each material were measured.

A Versa STAT V3-400 potentiostat (Ametek, Berwyn, PA, USA) was used for electrochemical tests. A classical three-electrode cell was used, with a Mg alloy specimen as the working electrode, a saturated calomel electrode (SCE) as the reference, and a platinum plate as the counter electrode. The SBF solution was used as a corrosion electrolyte. The scan rate for the polarization test was 1 mV/s. Cathodic and anodic polarization tests were carried out separately. The cathodic polarization tests were carried out after immersion at an open circuit for 10 s, 10 min, 3 h, 20 h, 3 days, 7 days, 10 days, and 14 days. The anodic polarization tests were carried out after immersion at an open circuit for 14 days. In situ optical images of the surface morphology of specimens were taken with a camera controlled by a computer after the polarization test.

### 2.4. Microscopic Corrosion Morphology Observation

To explore the corrosion mechanism of the studied alloys, the samples before and after removal of the corrosion products following immersion were characterized under a SEM (Zeiss Ultra 55) with EDS. After polishing the samples using the above-mentioned method, they were characterized after immersion in SBF solution for 10 s, 30 min, and 6 h, respectively. At room temperature, the samples were immersed in a chromic acid solution (180 g/L) for about 1–2 min to remove corrosion products, and then continued to be observed under a scanning electron microscope.

### 2.5. Mechanical Properties

The mechanical properties of the as-extruded bars were measured at a tensile speed of 1 mm/min on a universal Material Testing Machine (Shimazu AG-X Plus, Kyoto, Japan) at room temperature. The dog bone specimens with a gauge length of 30 mm, a width of 3mm, and a thickness 2.5 mm were used. At least three repeated tests for each condition were carried out.

## 3. Result

### 3.1. Microstructure

**Figure 1:**
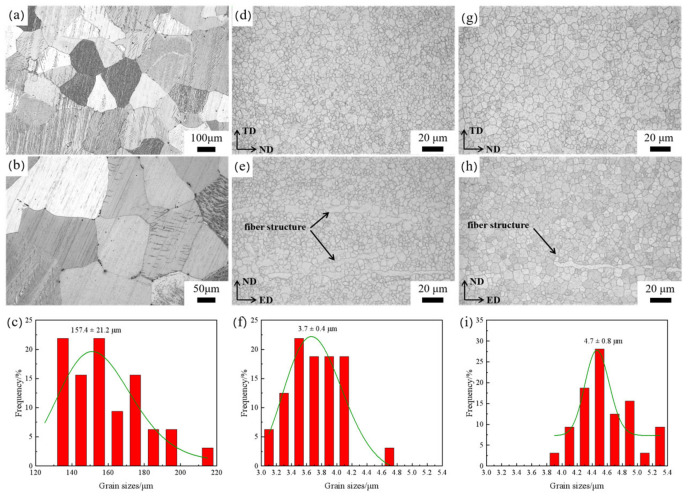
Optical microstructure and grain size distribution of the alloys: (**a**–**c**) HT-ZXK alloy; (**d**–**f**) 320-E alloy; (**g**–**i**) 350-E alloy. The green line represents the linear fitting outcome of the grain size distribution histogram.

Figure 1 shows the optical microstructure of the alloy under three conditions. At both extrusion temperatures, most of the grains of the alloy are recrystallized, and the recrystallization is more complete at 350 °C. A small amount of fibrous structure is included in both extruded alloys (Figure 1e,h). The average grain sizes of the HT-ZXK alloy, 320-E alloy, and 350-E alloy are 157.4 ± 21.2 μm, 3.7 ± 0.4 μm, and 4.7 ± 0.8 μm, respectively (Figure 1c,f,i), indicating that the grain sizes of homogenized alloys decrease sharply after extrusion. As the extrusion temperature increases, the grain size increases slightly.

**Figure 2:**
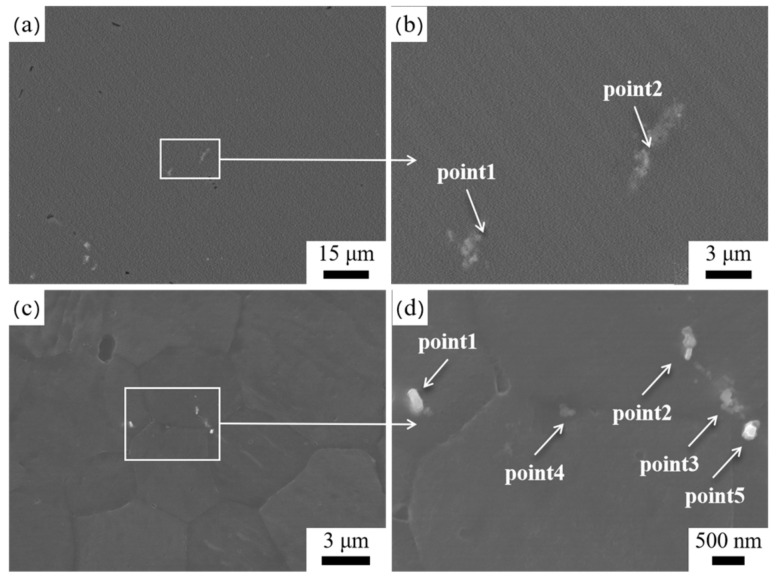
SEM images of the second phase in alloys: (**a**,**b**) HT-ZXK alloy; (**c**,**d**) 320-E alloy.

The SEM images in Figure 2 and the EDS results in Table 2 show that the second phase in the HT-ZXK alloy is mainly a Zr-rich phase containing a small amount of Fe, Al, and Si impurities, with a size of 3~5 μm and a granular shape. Under the condition of extrusion at 320 °C, the Zr-rich phase is significantly refined, less than 1 μm in size, irregular in shape, and randomly distributed in the alloy. The SEM image of the 350-E alloy is similar to that of the 320-E alloy.

### 3.2. Corrosion Properties

**Figure 3:**
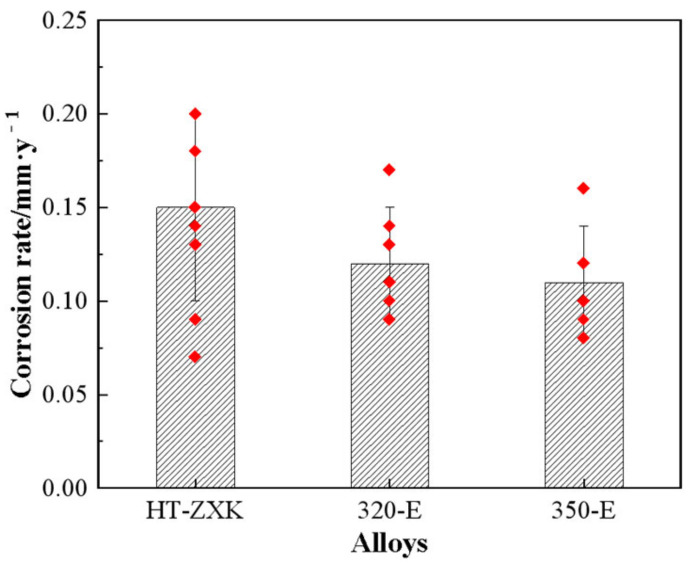
The corrosion rate of the studied alloys after immersion in SBF solution for 21 days, which is measured by mass loss. (The red dots represent the mass loss rate of parallel specimens).

Figure 3 shows the result of the mass loss of the investigated alloy immersed in SBF solution for 21 days. The results show that the average corrosion rates of the HT-ZXK alloy, 320-E alloy, and 350-E alloy are 0.15 mm/year, 0.12 mm/year, and 0.11 mm/year, respectively. The corrosion rate of the alloy is reduced by more than 25% after extrusion.

**Figure 4:**
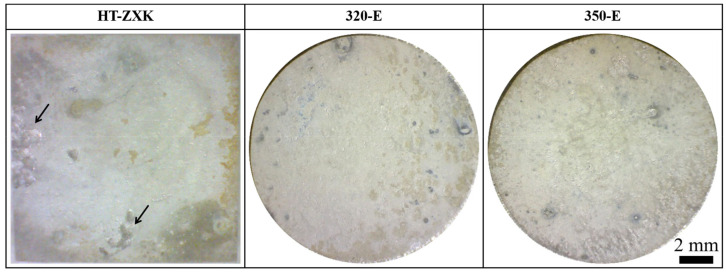
Optical images of the specimens after the mass loss test and removal of corrosion products.

Figure 4 shows the macroscopic corrosion morphology of the alloys after the mass loss test and removal of the corrosion products. Corrosion of the HT-ZXK alloy is the most severe, and the surface of the alloy has a wide range of local corrosion, as shown by the arrows. There is no obvious local corrosion in the extrusion alloys.

**Figure 5:**
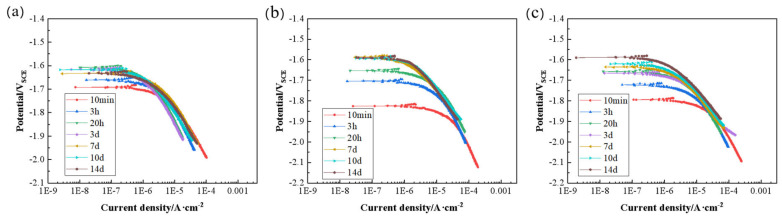
Cathodic polarization curves of the studied alloys after immersion in SBF for 10 min, 3 h, 20 h, 3 days, 7 days, 10 days, and 14 days: (**a**) HT-ZXK; (**b**) 320-E; (**c**) 350-E.

In order to study variation of the cathodic activity of the alloys, the cathodic polarization curve was measured after immersion in SBF solution for different times, as shown in Figure 5. As the corrosion time increased, the cathodic activity of the homogenized alloy fluctuated slightly, and the cathodic activity of the extruded alloy was relatively stable.

**Figure 6:**
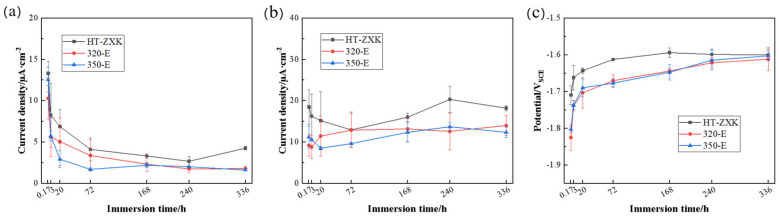
Cathode polarization parameters: (**a**) corrosion current density; (**b**) current density at −1.8 V_SCE_; (**c**) corrosion potential.

In order to better compare the electrochemical behavior of the alloys, the relevant parameters derived from the polarization curves were compared, as shown in Figure 6. During the 14-day immersion process, the corrosion current density of the three alloys showed a decreasing trend with extension of the immersion time, indicating that the corrosion rate of the alloys gradually decreased with an increase in immersion time. It can be clearly observed that the corrosion current density of the alloys decreased after extrusion. This indicates that the corrosion resistance of the alloys is improved after extrusion. There is no difference in the corrosion current density between 350-E and 320-E alloys, and the above results are consistent with the mass loss results (Figure 6a). The current density of the alloys at -1.8 V_SCE_ was taken to statistically compare cathode activity. The extrusion alloys always have the lowest cathodic activity and are more stable than the HT-ZXK alloys. There is little difference in cathode activity between the 320-E and 350-E alloys (Figure 6b). The lower corrosion potential indicates a greater corrosion tendency, and the corrosion potential of the HT-ZXK alloy is higher than that of the two extruded alloys (Figure 6c). Contrary to the actual corrosion rate results, it indicates that a high corrosion tendency does not mean a high corrosion rate.

**Figure 7:**
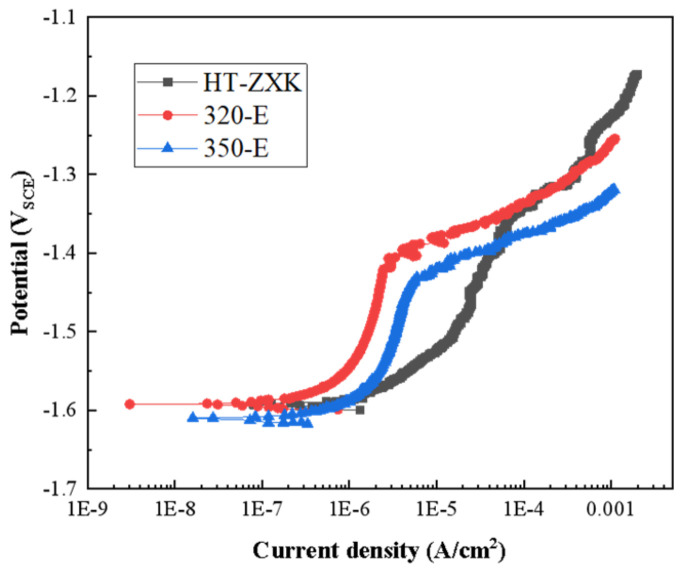
Anodic polarization curves of the studied alloys after immersion in SBF solution for 14 days.

To analyze the protective effect of the surface film formed during the immersion process of the studied alloy, we anodically polarized the alloy immersed for 14 days, as shown in Figure 7. The 320-E and 350-E alloys exhibited a clear breakdown potential, indicating that the surface film remained intact. However, no breakdown potential was observed for the HT-ZXK alloy, which indicates that the surface film of the alloy had been destroyed. Therefore, after extrusion, the surface film has a stronger protective effect on the magnesium matrix; this is due to the denser surface film of the extruded alloy.

**Figure 8:**
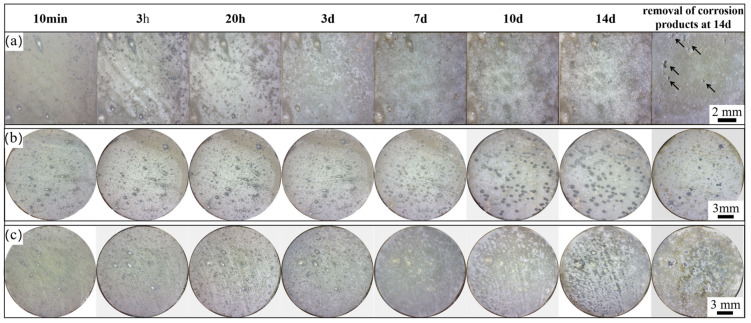
Optical images of the studied alloys after immersion in SBF solution for different times: (**a**) HT-ZXK alloy; (**b**) 320-E; (**c**) 350-E. (Arrows indicate localized corrosion sites).

Figure 8 displays the macroscopic corrosion morphology of the studied alloys. Cathodic active sites are clearly visible in all three alloys after a 10 min immersion. As the immersion time increases, the product coverage of the active site of the cathode also increases. At 7 days, the HT-ZXK alloy exhibited a dark corrosion site, which increased and expanded at 14 days (Figure 8a). The 320-E and 350-E alloys showed numerous dark corrosion sites at 10 days (Figure 8b,c). Upon removal of the corrosion products, the HT-ZXK alloy exhibited the most significant local corrosion pits, while no obvious corrosion pits were observed on the surface of the extruded alloys, as indicated by the arrows (Figure 8).

### 3.3. Microscopic Corrosion Morphology Analysis

**Figure 9:**
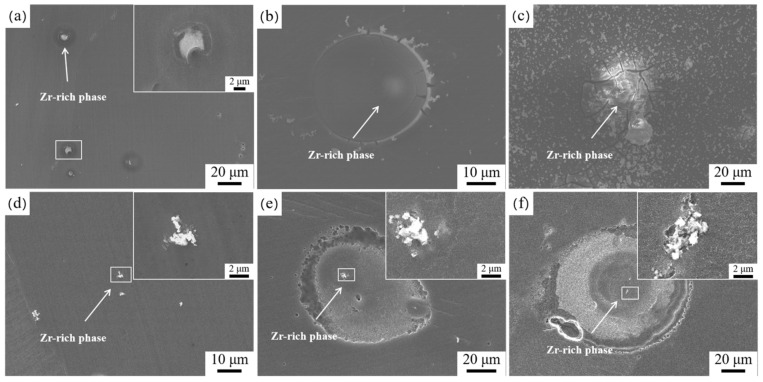
Micro-galvanic corrosion between Zr-rich phase and ɑ-Mg matrix in HT-ZXK alloy in SBF solution. Before removing corrosion products: (**a**) after immersion for 10 s; (**b**) after immersion for 30 min; (**c**) after immersion for 6 h. After removing corrosion products: (**d**) after immersion for 10 s; (**e**) after immersion for 30 min; (**f**) after immersion for 6 h.

**Figure 10:**
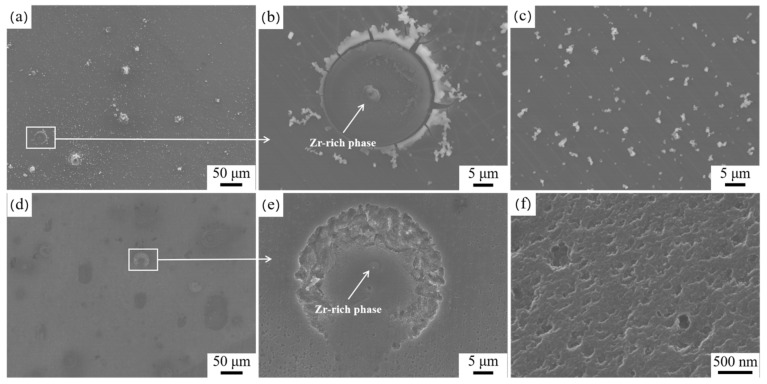
Micro-galvanic corrosion morphology between Zr-rich phase and ɑ-Mg matrix in 320-E alloy after immersion in SBF solution for 30 min. Before removing corrosion products: (**a**) low magnification; (**b**) Zr-rich phase; (**c**) magnesium matrix. After removing corrosion products: (**d**) low magnification; (**e**) Zr-rich phase; (**f**) magnesium matrix.

To compare galvanic corrosion behavior between the Zr-rich phase and magnesium matrix before and after extrusion of the alloy, microscopic corrosion behavior was observed by SEM. Figure 9 shows the micromorphologies of the HT-ZXK alloy immersed for different times. When immersed for 10 s, the Zr-rich phase was covered by a small amount of corrosion products (Figure 9a). After removal of the corrosion products, no significant corrosion was observed (Figure 9d). After immersion for 30 min, the corrosion products increased, and both the substrate and the Zr-rich phase were covered (Figure 9b). After removing the corrosion products, it is observed that annular corrosion occurs around the Zr-rich phase, while corrosion near the Zr-rich phase is lighter. After immersion for 6 h, the increase in corrosion products on the Zr-rich phase was most obvious, and the corrosion products were significantly higher than the magnesium matrix (Figure 9c). After removal of the corrosion products, annular corrosion became severe, and light corrosion occurred near the Zr-rich phase (Figure 9f). Figure 10 shows the microscopic corrosion behavior of the extruded alloy immersed in SBF solution for 30 min. Similarly, the Zr-rich phase is also covered by more corrosion products (Figure 10a,b), and the magnesium matrix is uniformly covered by corrosion products (Figure 10c). After removal of the corrosion products, it can be observed that annular corrosion near the Zr-rich phase is more obvious (Figure 10d,e), and corrosion of the magnesium matrix is more uniform and less corroded (Figure 10f).

### 3.4. Mechanical Properties

**Figure 11:**
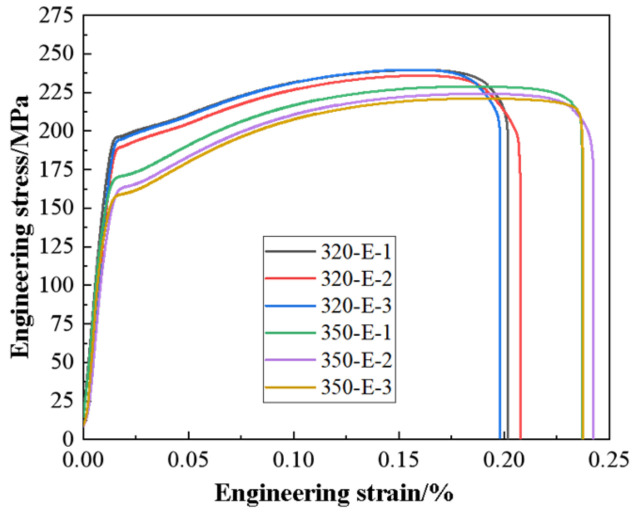
Engineering stress–strain curves of as-extruded alloys at room temperature.

Figure 11 displays the tensile engineering stress–strain curve at room temperature for two extrusion temperatures. Low-temperature extrusion of the 320-E alloy exhibits a high engineering stress of approximately 188.8 MPa for yield strength, 236.9 MPa for ultimate tensile strength, and 20.2% for elongation. As the extrusion temperature increases, the yield strength of the 350-E alloy decreases to 163.4 MPa, the ultimate tensile strength to 225.2 MPa, and elongation to 22.9%.

**Figure 12:**
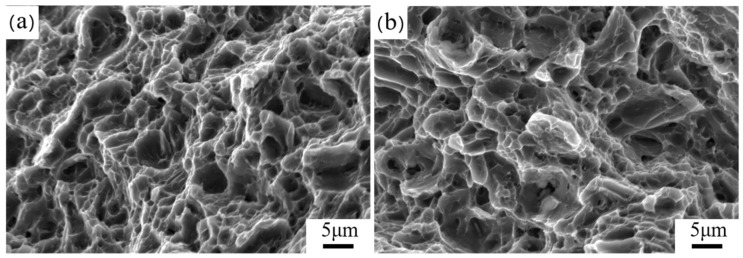
Images of fracture morphology of the as-extruded alloys: (**a**) 320-E; (**b**) 350-E.

Figure 12 shows the fracture morphology of the samples after tensile tests. Typical ductile cracks with pits were found in both alloys at both extrusion temperatures, indicating a ductile fracture mode.

## 4. Discussion

### 4.1. Phase Formation

**Figure 13:**
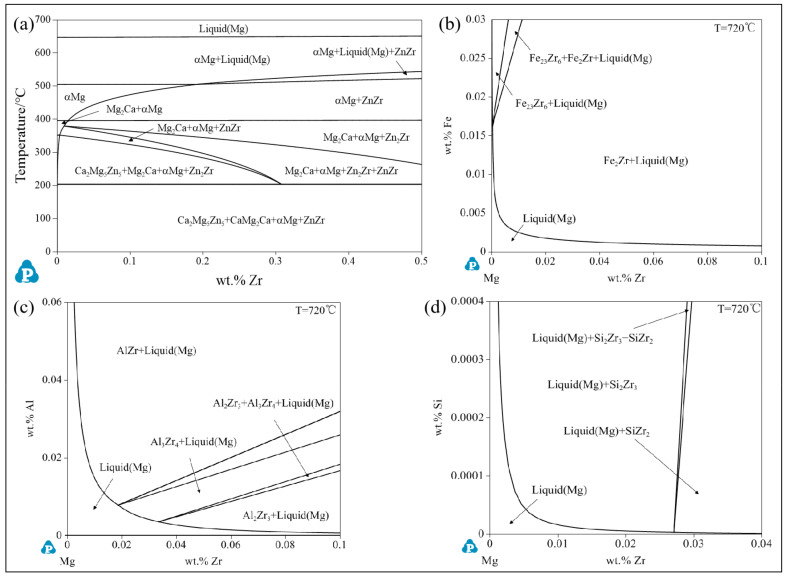
Phase diagram calculated using Pandat software: (**a**) Mg–0.5Zn–0.3Ca–xZr (wt.%) phase diagram at the Mg-rich corner; (**b**–**d**) are the isothermal cross-sections of the ternary phase diagrams for Mg–Fe–Zr, Mg–Al–Zr, and Mg–Si–Zr at 720 °C, respectively.

Based on the calculated phase diagram in Figure 13a, the HT-ZXK alloy should be composed of ɑ-Mg only. However, a Zr-rich phase containing a small amount of Fe, Al, and Si was found. It can be seen from (b), (c), and (d) in Figure 13 that Zr can form Zr_x_Fe_y,_ Zr_x_Al_y_, and Zr_x_Si_y_ phases with Al and Si [46,47]. However, Zr content in the Zr-rich phase in the present work is much higher than Zr content in the Zr_x_Fe_y,_ Zr_x_Al_y_, and Zr_x_Si_y_ phases shown in the calculated phase diagrams. Thus, the Zr-rich phase might be undissolved Zr particles from the added Mg–Zr master alloy, and trace impurities of Fe, Al, and Si only reacted with the surface of the Zr-rich particles. Qian et al. [48,49,50] also demonstrated that irregularly shaped undissolved zirconium particles in magnesium alloys can contain a small amount of iron impurities, and the atomic ratio of Zr/Fe in these particles is much higher than that of Zr–Fe intermetallic compounds.

### 4.2. Influence of Zr-Rich Phase on Corrosion Behavior

The Zr-rich phase is detrimental to the corrosion resistance of magnesium alloys. The voltage potential of the Zr-rich phase is approximately 426 mV higher than that of the magnesium matrix [26,51,52,53]. The potential difference between the Zr-rich phase and the magnesium matrix in this study may be greater due to the presence of impurity elements in the Zr-rich phase. From a thermodynamic perspective, a high voltage potential difference can provide a strong driving force for micro-galvanic corrosion. Thus, the Zr-rich phase can accelerate corrosion of the magnesium matrix. Most studies have shown that solute Zr atoms increase the corrosion rate by enhancing anodic dynamics by changing the properties of the corrosion product film [54,55,56,57]. However, this effect of Zr was not found in the present work, which is probably because the Zr amount is too low.

**Figure 14:**
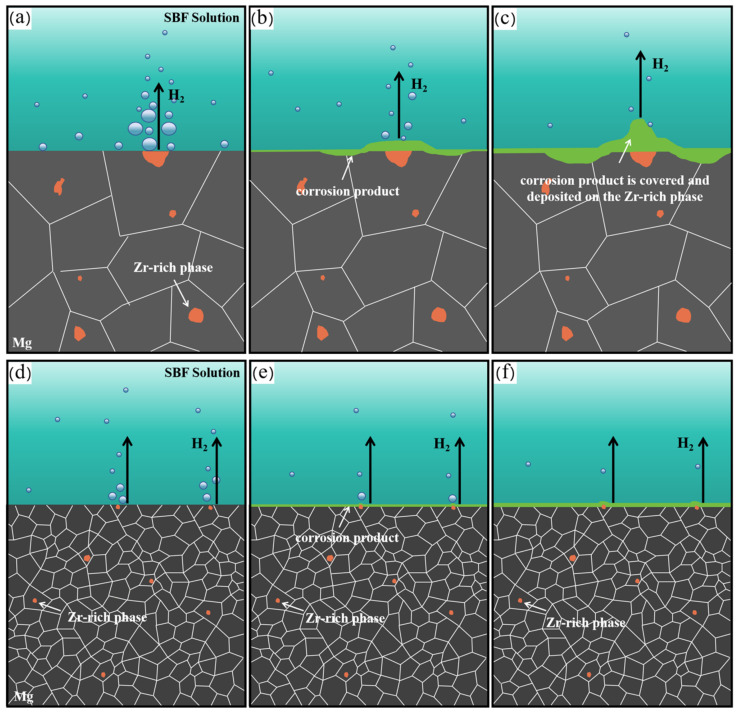
Schematic diagram of corrosion mechanism of Zr-rich phase: (**a**) initial corrosion behavior of HT-ZXK alloy; (**b**) continuous corrosion behavior of HT-ZXK alloy; (**c**) further corrosion behavior of HT-ZXK alloy; (**d**) initial corrosion behavior of as-extruded alloy; (**e**) continuous corrosion behavior of as-extruded alloy; (**f**) further corrosion behavior of as-extruded alloy.

Figure 14 shows a schematic diagram of the corrosion mechanism. With an increase in corrosion time, the magnesium matrix gradually dissolves. The Zr-rich phase is the main cathode active site. Hydrogen evolution reactions occur on the Zr-rich phase, and the produced OH^-^ anions continuously combine with the nearby Mg^2+^ to form Mg(OH)_2_ and deposit on the Zr-rich phase. The deposited corrosion product film can inhibit the cathodic activity of the Zr-rich phase to a certain extent, which depends on the size of the Zr-rich phase. For example, as shown in Figure 14d–f for extruded alloys, because the Zr-rich phase is refined, the corrosion product is more likely to cover and deposit on the Zr-rich phase; the cathodic activity is reduced, and the corrosion resistance of the extruded alloy is improved. Many studies have shown that the corrosion resistance of magnesium alloys can be improved by refinement of the second phase [58,59,60]. In addition, a decrease in grain size will also reduce the corrosion rate of magnesium alloy [61,62] because as the number of grain boundaries increases, the denser the surface corrosion film and the stronger the adhesion [35,63].

## 5. Conclusions

The effect of the extrusion process on the microstructure, corrosion properties, and mechanical properties of Mg–0.5Zn–0.3Ca–0.03Zr alloy has been studied in the present work. Conclusions can be drawn as follows:

1. In addition to a small amount of Zr-rich phase, no other precipitated phase was detected in the homogenized alloy. After extrusion at 320 °C and 350 °C, the size of Zr-rich particles was significantly reduced, and the extrusion temperature has little effect on the size of Zr-rich particles. However, the mechanical properties of the alloy decrease with an increase in extrusion temperature.
2. The corrosion rate of the alloy decreases after extrusion. This is because the smaller Zr-rich phase is easier to cover by the deposited corrosion products, which reduced the cathodic activity of the Zr-rich phase in the extruded alloys. Additionally, a much smaller grain size can promote the formation of a more uniform corrosion film on the surface of the extruded alloys.
3. The alloy extruded at 320 °C shows good comprehensive mechanical properties. The alloy extruded at 320 °C possesses excellent comprehensive mechanical properties and corrosion resistance. Specifically, the yield strength (TYS) amounts to 188.8 MPa; the elongation (EL) reaches 20.2%; and the corrosion rate is 0.12 mm/year. These properties are anticipated to enable its application in the domain of medical biological magnesium alloys.

## Figures and Tables

**Table 1:** Compositions of the magnesium alloys studied in the present work (wt%).

Sample	Zn	Ca	Zr	Fe	Ni	Si	Mg
Mg–0.5Zn–0.3Ca–0.05Zr	0.461	0.272	0.034	0.0029	0.00021	<0.00001	Balance

**Table 2:** EDS result statistics of the second phase of the alloy in Figure 2. (wt.%).

Alloys	Precipitates	O	Mg	Zn	Ca	Zr	Fe	Al	Si
HT-ZXK	Point1	5.12	81.51	0.97	0.08	11.44	0.25	0.10	0.53
	Point2	6.50	77.98	1.06	0.22	13.95	0.19	-	0.21
320-E	Point1	6.29	74.71	1.17	0.28	15.03	0.37	1.68	0.46
	Point2	5.72	90.64	0.77	0.24	2.44	-	-	0.62
	Point3	11.39	66.67	1.05	0.38	17.26	0.63	1.97	0.65
	Point4	10.35	78.44	1.16	0.29	9.50		0.17	0.08
	Point5	5.51	75.78	0.64	0.50	15.35	0.32	0.48	1.42

## Data Availability

The raw/processed data required to reproduce these findings cannot be shared at this time because the data also form part of an ongoing study.
